# Pulse-Driven MEMS NO_2_ Sensors Based on Hierarchical In_2_O_3_ Nanostructures for Sensitive and Ultra-Low Power Detection

**DOI:** 10.3390/s24227188

**Published:** 2024-11-09

**Authors:** Haixia Mei, Fuyun Zhang, Tingting Zhou, Tong Zhang

**Affiliations:** 1 Key Lab Intelligent Rehabil & Barrier Free Disable (Ministry of Education), Changchun University, Changchun 130022, China; meihx@ccu.edu.cn; 2 State Key Laboratory of Integrated Optoelectronics, College of Electronic Science and Engineering, Jilin University, Changchun 130012, China; zhangfy2021@163.com

**Keywords:** MEMS, gas sensor, NO_2_, high sensitivity, ultra-low power consumption

## Abstract

As the mainstream type of gas sensors, metal oxide semiconductor (MOS) gas sensors have garnered widespread attention due to their high sensitivity, fast response time, broad detection spectrum, long lifetime, low cost, and simple structure. However, the high power consumption due to the high operating temperature limits its application in some application scenarios such as mobile and wearable devices. At the same time, highly sensitive and low-power gas sensors are becoming more necessary and indispensable in response to the growth of the environmental problems and development of miniaturized sensing technologies. In this work, hierarchical indium oxide (In_2_O_3_) sensing materials were designed and the pulse-driven microelectromechanical system (MEMS) gas sensors were also fabricated. The hierarchical In_2_O_3_ assembled with the mass of nanosheets possess abundant accessible active sites. In addition, compared with the traditional direct current (DC) heating mode, the pulse-driven MEMS sensor appears to have the higher sensitivity for the detection of low-concentrations of nitrogen dioxide (NO_2_). The limit of detection (LOD) is as low as 100 ppb. It is worth mentioning that the average power consumption of the sensor is as low as 0.075 mW which is one three-hundredth of that in the DC heating mode. The enhanced sensing performances are attributed to loose and porous structures and the reducing desorption of the target gas driven by pulse heating. The combination of morphology design and pulse-driven strategy makes the MEMS sensors highly attractive for portable equipment and wearable devices.

## 1. Introduction

MOS gas sensors are widely used in numerous fields due to the advantages of high sensitivity, fast response, wide range of gas concentration sensing, easy fabrication and low cost [1,2]. However, as typical semiconductor gas sensors, they are usually required to operate at about 100–450 °C [3,4] because high temperature is needed to realize the intrinsic excitation of MOS materials, which makes conductive electrons of the material surface easier to be captured, and causes different chemisorption oxygen to be formed, thus producing sensitive properties [5]. Heating resistors are always used to provide the operating temperature for sensitive materials, resulting in high energy consumption and limited practical applications in mobile and portable devices [6]. How to reduce the power consumption of MOS sensors and provide more possibilities for application scenarios has become an inevitable problem.

Researchers have made a lot of attempts in different directions to cut down the power consumption of MOS gas sensors. One obvious and effective method to cut down power consumption is to decrease the working temperature by changing the morphology, composition and structure of the sensitive materials [7,8]. A reported Fe_2_(MoO_4_)_3_ of square flower-shaped microporous with a layered structure was developed in ethanol sensing which could work at 140 °C with a sensitivity of 68.5 for 100 ppm ethanol [9]. A Ni-doped ZnO sensor decorated with the optimum concentration of reduced graphene oxide (rGO) was reported, which showed the maximum sensing response of about 63.8% for 100 ppm hydrogen at 150 °C [10]. The sensing material rGO/Cu_2_O nanocomposites which were synthesized by a water-bath heating method showed a very low detection limit of 1 ppb NH_3_, a fast response/recovery rate (1/1 s to 5 ppb NH_3_) and excellent selectivity to 5 ppb NH_3_ at 80 °C temperature [11]. However, the reduction in power consumption due to temperature reduction was limited and MOS gas sensors still had a low sensitivity or slow response recovery at the ambient temperature, so more explorations are needed in the new ways for power consumption reduction.

**Figure 1:**
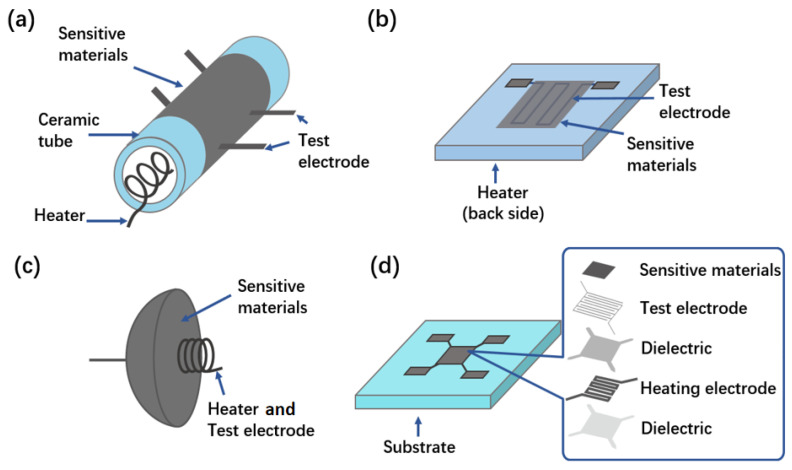
Four typical MOS gas sensor structures. (**a**) Ceramic tube gas sensor. (**b**) Ceramic chip gas sensor. (**c**) Hot-wire gas sensor. (**d**) MEMS gas sensors.

Adopting new gas sensor structures is another effective way to reduce power consumption. Researchers have developed various gas sensors based on different heating platforms; four typical structures sensors are investigated in this work and the structure diagrams are shown in Figure 1. The most classical cylindrical ceramic tube structure gas sensor (see Figure 1a) has one pair of spiral heating wire inside, two pairs of test electrodes outside and sensitive material is coated on the surface. The power consumption of gas sensors based on the cylindrical ceramic tube was about 800 mW [12]. The planar ceramic gas sensor (as shown in Figure 1b) has a platinum wire heater in the back and a test electrode in the front, and the sensitive material is the coating on the front surface [13]. The power consumption of the ceramic plate gas sensors was about 500 mW [14]. The hot-wire gas sensor (see Figure 1c) is a round beaded device made by coating the material on the platinum wire coil, which is not only a heating electrode but also a test electrode. The power consumption of the hot-wire gas sensors was about 150–300 mW [15,16]. With the development of the silicon technique and the appearance of a micro heater, MOS gas sensor gradually developed towards miniaturization, easy integrated circuit integration and low consumption [17]. MEMS gas sensors (as shown in Figure 1d) usually consist of four main components: sensitive materials, test electrode, dielectric and a heating electrode [18]. MEMS gas sensors can further reduce the power consumption to as low as a few milliwatts compared with gas sensors based on cylindrical ceramic tube structures and flat ceramic structures [19]. Xie et al. reported a novel metal oxide semiconductor (MOS) gas sensor based on a single cantilever, which showed a good linear characteristic to 0–10 ppm ethanol and a power consumption of 2.96 mW [20]. Kim et al. reported a batch nanofabrication method of a suspended single 1D nanoheater for a gas sensor with a work temperature of 250 °C and power consumption of 1.62 mW [21]. The reasons for the power consumption reduction were sensor miniaturization and structure enhancement. However, all these are limited by the manufacturing accuracy of integrated circuits, and the power consumption will enter a bottleneck period by cutting the micro heater size. There is still great research significance to develop new methods to further reduce the power consumption of gas sensors [22,23].

The survey found that pulse heating can effectively reduce the power consumption of gas sensors by cutting down the average power consumption during operation [24,25]. Zettl and Mickelson et al. optimized the MEMS structure of the sensor to realize that the power consumption of the sensor is only 2.1 mW during continuous operation at 300 °C [26]. At the same time, pulse heating also has some unique characteristics in sensor performance, such as the realization of lower detection limit, multi-gas detection and gas recognition [27,28]. The previous work also found that the sensor performance decreased when the power consumption cut down under the pulse heating mode. Palacio et al. used sensors (fabricated in the Institute for Microelectronics and Microsystems, Italy) to explore the behaviors of Pulsed-Temperature Operation (PTO) metal oxide gas sensors, which reached power savings of 99% and resulted in a moderate decrease in sensor sensitivity [29]. 

At the same time, to meet the detection requirement of trace target gases in complex environments and miniaturized, portable devices, the gas sensors with the lower limit of detection (LOD) and power consumption are necessary [30,31]. The response signals of the MOSs’ gas sensors arise from the reaction between adsorbed oxygen and target gases on the surfaces of sensing layers at the relatively high operating temperature [32,33]. Thus, the ability of oxygen adsorption is very important for the sensing materials. Hierarchical nanostructures are assembled from low dimensional nano-building blocks, exhibiting a less agglomerated configuration, well-aligned porous structure and high surface area [34,35]. The unique structure can provide accessible gas diffusion and more active sites for sensing reactions [36]. For example, Chen et al. prepared the flower-like Zn_2_SnO_4_ three-dimensional (3D) hierarchical nanostructures using a simple one-step hydrothermal method. The response to 50 ppm of ethanol was 30.4 for the sensor based on the flower-like Zn_2_SnO_4_, while the response of the sensor based on Zn_2_SnO_4_ with the dense structure was only 12.8 [37]. Fan et al. synthesized hierarchical Pt/SnO_2_ nanosheets using the hydrothermal method. The sensor based on 2.0% Pt/SnO_2_ exhibited a high response (27.85) to 100 ppm ethanol gas [38]. Du et al. prepared the hierarchical NiCo_2_O_4_ hollow microtubules. Results show that the response of hierarchical NiCo_2_O_4_ hollow microtubules was 9.2, which was higher than the NiCo_2_O_4_ nanosheets. The LOD of the sensor based on NiCo_2_O_4_ hollow microtubules was 1 ppm [39].

NO_2_ is one pollution gas that can cause harm to human health and the environment [40,41]. Considering the detection requirement in trace NO_2_ gases in complex environment and miniaturized, portable devices, the gas sensors with the lower limit of detection (LOD) and consumption are necessary. In this work, the uniform indium oxide (In_2_O_3_) hierarchical materials that were assembled with two-dimensional (2D) thin nanosheets were prepared via a hydrothermal method followed by heating treatment. The MEMS gas sensors were fabricated using synthesized hierarchical In_2_O_3_ as the sensing materials. In order to improve the sensing performance and reduce the sensor power, a rational pulse-driven method for MEMS gas sensors was employed. Compared with DC heating, the response of the sensor to different concentrations (0.1~4 ppm) of nitrogen dioxide (NO_2_) in pulse-driven mode was significantly increased. The LOD was reduced from 1 ppm (DC) to 100 ppb (pulse-driven method). In addition, the power consumption for pulse-driven MEMS gas sensors was as low as 0.075 mW. Finally, the physical–chemical adsorption process of the gas molecules onto the MOSs surface was analyzed in the pulse-driven mode, providing a suitable method for the MEMS gas sensors with a high performance and ultra-low power consumption in future work.

## 2. Materials and Methods

### 2.1. MEMS NO_2_ Gas Sensor

In this study, hierarchical In_2_O_3_ materials were synthesized with slight alterations on the basis of previous research [42]. First, a mixed solution was prepared by dissolving 0.489 g of indium chloride (InCl_3_·4H_2_O) and 1.363 g of sodium dodecyl sulfonate (SDS) in 100 mL deionized (DI) water. Subsequently, 0.5 g of urea (CO(NH_2_)_2_) was introduced into the aforementioned solution and stirred for 2 h. The hydrothermal reaction of the mixed solution was performed at 120 °C for 9 h using a Teflon-lined reactor. The products were then centrifuged and rinsed with DI water and ethanol after naturally cooling down to the ambient temperature. Then, the mixture was calcined at 500 °C for 2 h to obtain the yellow In_2_O_3_ materials.

**Figure 2:**
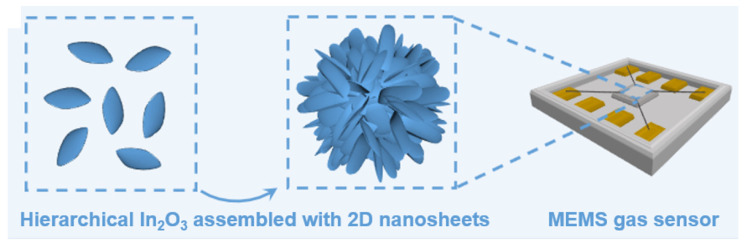
Schematic diagram of the formation of the hierarchical In_2_O_3_ and the MEMS sensor.

To prepare the MEMS gas sensors, as-obtained yellow powder was added to a mixture of ethanol and DI water to form a homogeneous paste. Next, the paste was evenly coated on the MEMS Micro-Hot-Plate (MHP). The MHP chip (INS-100) was purchased from Suzhou Xinmeixin Electronic Technology Co (Suzhou, China). The MHP chip size was 1.0 × 1.0 × 0.3 mm, equipped with a pair of heating electrodes and interdigital electrodes. The heating area was 0.3 × 0.3 mm. To form the stable sensing layer, the prepared MEMS sensors were dried in the oven at 60 °C for 6 h. In addition, the gas sensors were aged at 300 °C for 24 h before gas-sensing measurements. The schematic diagram of the formation of the hierarchical In_2_O_3_ and the MEMS sensor is shown in Figure 2.

### 2.2. The Test System

**Figure 3:**
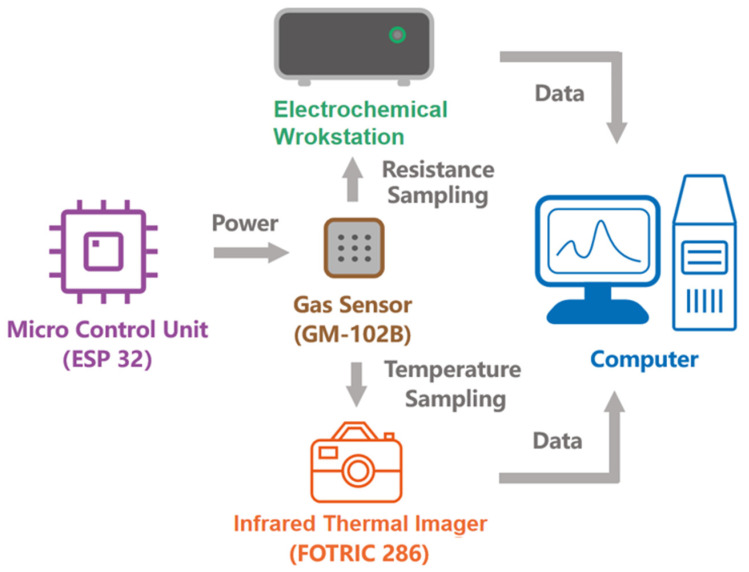
The structure diagram of the measuring system used for the NO_2_ detection.

Aiming at the dynamic temperature and resistance characteristics of MEMS MOS sensor, a test system was designed and implemented with the structure diagram shown in Figure 3. The system consisted of a Microprogrammed Control Unit (MCU), infrared imaging device, electrochemical workstation and computer.

MCU (ESP32) provided a continuous or pulse-heating voltage for the sensor. The embedded digital-to-analog converter (DAC) in the ESP32 generated a voltage control signal and a voltage follower to ensure the output current reached a relatively high value to form the heating voltage which was applied on the heating electrodes.

Infrared imaging device (FOTRIC 286), which has the spatial resolution of 1.14 mrad and the range of temperature measurement from −40 °C to 700 °C, captured the surface temperature of sensitive material in real time and transmitted the temperature data to the computer for display.

Electrochemical workstation was used to realize the accurate measurement of the dynamic sensitive resistance, and the sensing data were analyzed and displayed on the computer too.

### 2.3. Experiments

The structure diagram of the measuring system for NO_2_ detection is shown in Figure 3. According to the sensor’s characteristics, the sensor needs to be preheated with a continuous heating voltage for 72 h to improve its stability before being used, and MCU ESP32 supplied the heating voltage for the sensor. The parameters of the heating voltage, including voltage amplitude, the cycle’s length T*_cycle_*, the heating time T*_on_*, the waiting time T*_wait_* and the duty ratio, which is defined by T*_on_*/T*_cycle_*, could be regulated through programming control in ESP32.

In continuous heating, a programing continuous voltage was applied on the sensor as the heating voltage, and the response of the MEMS sensor in different NO_2_ concentrations (from 100 ppb to 4 ppm) was tested.

In pulse-heating mode, the parameter T*_on_* was adjusted from 1 s to 0.1 s to survey the fast thermal response of the micro heater in the MEMS gas sensor, and then a heating cycle voltage (T*_on_* = 0.1 s, T*_wait_* = 29.9 s) was supplied as the heating voltage and the sensitive characteristics of the MEMS sensor in the air and different NO_2_ gas concentrations (from 100 ppb to 4 ppm) was studied.

## 3. Results and Discussion

### 3.1. Material Structural Characterization

**Figure 4:**
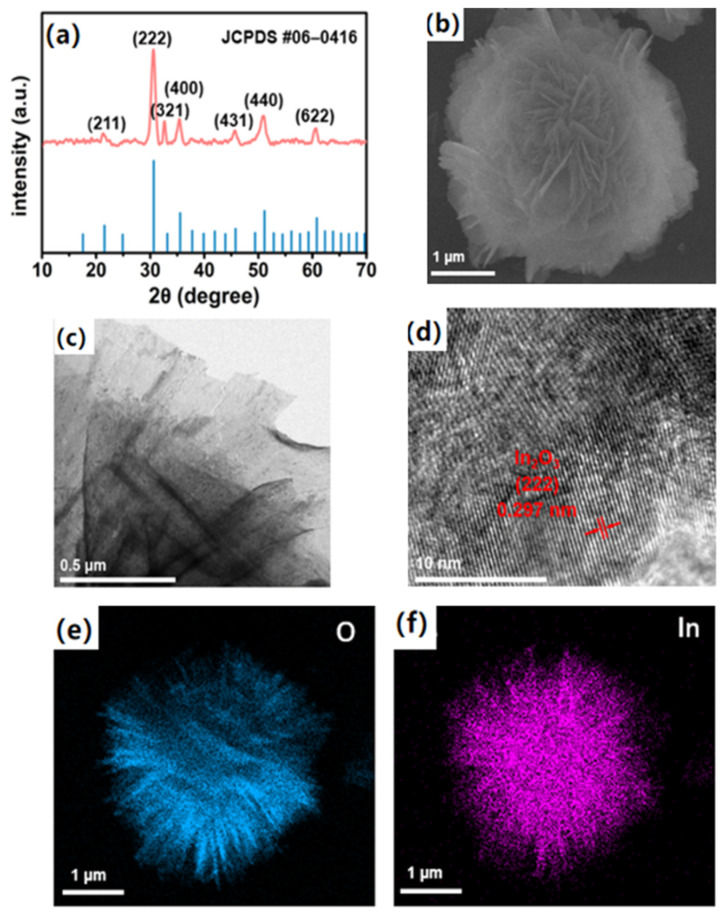
(**a**) XRD pattern; (**b**) SEM image; (**c**) TEM image; (**d**) HRTEM image; (**e**,**f**) EDS mapping images for O and In of In_2_O_3_.

The X-ray diffraction patterns (XRD) of the material were performed to analyze the structure and crystal phases of the obtained materials, and the result is shown in Figure 4a, where the diffraction peaks match well with the cubic phase of In_2_O_3_ (JCPDS No. 06-0416). The high intensity of the diffraction peaks indicates that the In_2_O_3_ samples had good crystallinity. The morphologies and structural characteristics of the prepared In_2_O_3_ were investigated through scanning electron microscopy (SEM) and the transmission electron microscope (TEM). The SEM image in Figure 4b shows that the hierarchical In_2_O_3_ was assembled by a mass of nanosheets. The analyses of the TEM image in Figure 4c were further employed to observe the detailed morphological characteristics. As shown in Figure 4c, the sample had a monodisperse uniform 3D structure with thin and loose 2D nanosheets. From the high resolution transmission electron microscope (HRTEM), the lattice fringe (ca. 0.297 nm) marked on the image corresponded to (222) plane of cubic phase In_2_O_3_ (Figure 4d). The element composition of the samples was analyzed via the spectrum of energy dispersive spectroscopy (EDS). From the EDS mapping images in Figure 4e,f, O and In could be found in the final products and were evenly dispersed in the material.

### 3.2. Temperature Characteristics of the MHP

**Figure 5:**
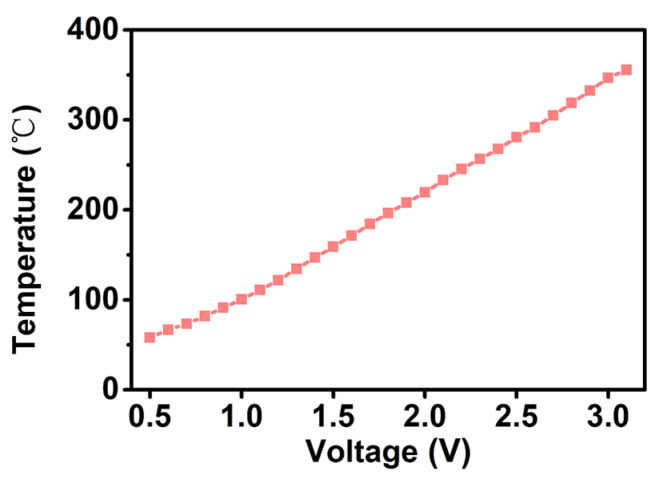
The surface temperature variation in In_2_O_3_ sensors with different DC heating voltages.

The temperature characteristics of sensitive materials on the MHP with the DC heating voltage were investigated because MOS sensing materials always need to work at a high temperature. The infrared emission coefficient of the MHP was set at 0.3, which was the same value used in the temperature measurement. Through the established measuring system, the surface temperature of the MHP after coating the materials was linear with the heating voltage, as shown in Figure 5.

### 3.3. DC Heating Mode

**Figure 6:**
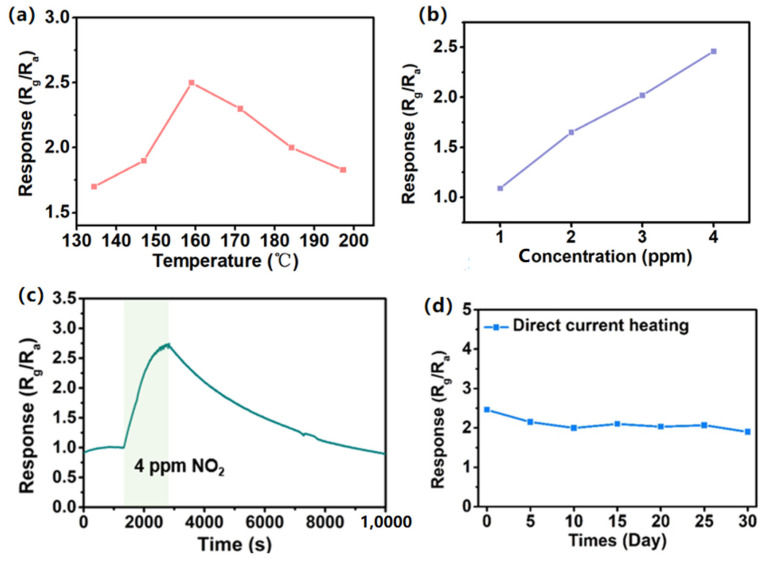
In_2_O_3_ sensors under DC heating mode. (**a**) The temperature variation in the MHP with different voltages. (**b**) Responses of the sensor to different concentrations of NO_2_. (**c**) Dynamic response curves of the sensor based on hierarchical In_2_O_3_ under the DC heating mode for 4 ppm NO_2_. (**d**) The long-term stability to the sensor on based hierarchical In_2_O_3_ for 4 ppm NO_2_ under the DC heating mode.

To investigate the optimum operating temperature for the hierarchical In_2_O_3_, the gas responses were measured from 135 to 195 °C with a heating voltage of 1.3–1.9 V. The corresponding relationship between temperature and the different sensors responses to 4 ppm NO_2_ is illustrated in Figure 6a. The sensor response under DC heating mode was defined as the ratio of the resistance in NO_2_ (Rg) to the resistance in air (Ra). For the sensor based on hierarchical In_2_O_3_, the highest response (R_g_/R_a_ = 2.5) was obtained at 160 °C, and the heating power consumption was 22.5 mW. Figure 6b indicates the sensor responses to different concentrations of NO_2_, which shows good linearity. The response/recovery curve of the sensor based on hierarchical In_2_O_3_ to 4 ppm NO_2_ at 160 °C is shown in Figure 6c. At DC heating mode, the LOD was 1 ppm for the sensor based on hierarchical In_2_O_3_ and had no response to NO_2_ with the lower concentrations. The long-term stability of the hierarchical In_2_O_3_ sensor for 4 ppm NO_2_ under the DC heating mode is shown in Figure 6d.

### 3.4. Pulse-Driven Mode

The pulse-driven voltage used in this research was set to 1.5 V at which the sensing material could reach the optimum temperature. The temperature values that the sensitive material surface reached under different pulse-heating voltages were compared. A heating cycle time T*_cycle_* included heating time T*_on_* and waiting time T*_wait_*, and whether ultra-low power consumption could be realized depended on the reduction in average power consumption P*_average_* which was a part of the continuous heating power consumption P*_C_* and calculated by P*_average_* = P*_C_* × T*_on_*/T*_cycle_*.

**Figure 7:**
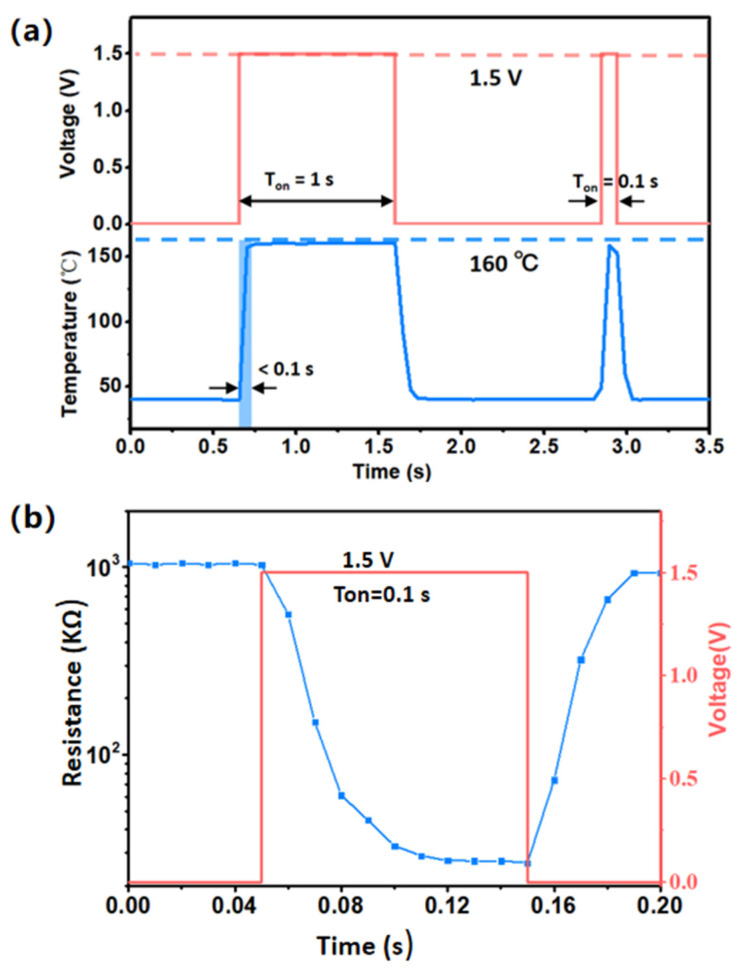
Sensor on based hierarchical In_2_O_3_ under the pulse-driven mode. (**a**) Temperature characteristics of the MHP. (**b**) The sensitive characteristics of the sensor under the pulse-driven mode.

At first, one pulse-heating cycle voltage (T*_on_* = 1 s, T*_wait_* = 1 s) was supplied to the MHP, and it can be seen from Figure 7a that the temperature quickly reached the optimum temperature 160 °C at T*_on_* beginning and sharply decreased to normal temperature when T*_on_* ended. The quick response time of temperature was about 0.1 s, so another pulse-heating cycle voltage (T*_on_* = 0.1 s, T*_wait_* = 1 s) was applied to further investigate. The experiment shows the temperature could reach up to 160 °C in 0.1 s too. It was found that the accurate response time was less than 0.1 s and recovery time was about 0.1 s from the temperature response curve with the time axis extended. No further precise data can be given due to the sampling time limitations of the infrared imaging device. From Figure 7a, we can draw the conclusion that the sensor can reach its optimum temperature within 0.1 s working voltage based on the quick response characteristic of the MHP, and the temperature can be held until the moment when the applied voltage is removed.

On this basis, we chose a pulse-heating voltage (T_on_ = 0.1 s, T_wait_ = 29.9 s) to analyze the sensitive characteristics of the sensor under the pulse-driven mode. The resistance change characteristics of the sensor are shown in Figure 7b. At the beginning of the T_on_, the hierarchical In_2_O_3_ sensor resistance was rapidly reduced and then maintained. When the heating of the sensor was stopped, the resistance of the sensor rose rapidly. In this work, the terminal resistances of each T_on_ were extracted from the measured resistance waveform.

**Figure 8:**
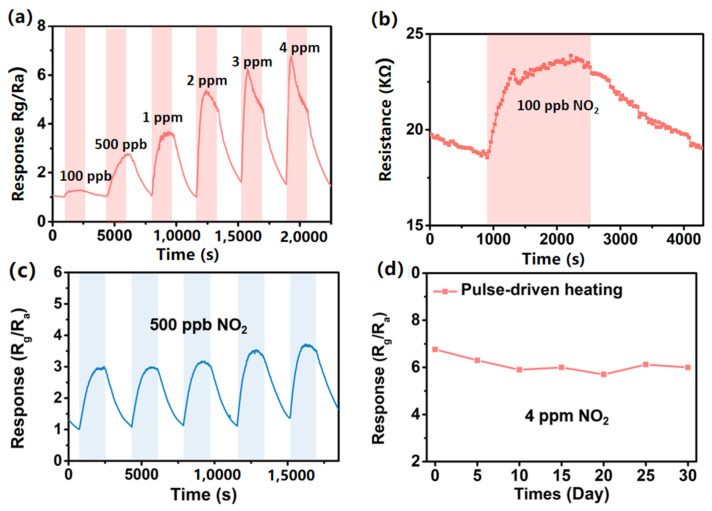
Sensor based on hierarchical In_2_O_3_ under pulse-driven heating mode: (**a**) dynamic response curves of sensor based on hierarchical In_2_O_3_ for 0.1–4 ppm NO_2_; (**b**) dynamic response curves of sensor based on hierarchical In_2_O_3_ for 0.1 ppm NO_2_; (**c**) the dynamic response curves of the sensor for 500 ppb NO_2_ during five cycles; (**d**) long-term stability.

The sensor response under the pulse-driven heating mode was defined as the ratio of the terminal resistance in NO_2_ (R_g_) to the terminal resistance in air (R_a_). The dynamic response curves of different concentrations of NO_2_ were acquired as depicted in Figure 8a. It can be seen clearly that the sensor based on hierarchical In_2_O_3_ possesses response–recovery sensing characteristics over the whole testing concentration ranging from 100 ppb to 4 ppm under the pulse-driven heating mode. The LOD of the sensor were reduced from 1 ppm in the DC heating mode to 100 ppb in the pulse-driven mode as shown in Figure 8b. The response and recovery times of the sensor to 100 ppb NO_2_ were 900 s and 1380 s, respectively. The responses of the sensor based on hierarchical In_2_O_3_ in different heating modes are given in Figure 8c. Compared with DC heating modes, the sensor working in the pulse-driven heating mode showed the higher sensitivity.

The average power consumption (P_average_) in the pulse-driven heating mode was calculated by the formula [26] as follows:

P_average_ = PC × T_on_/T_cycle_ (1)

Herein, PC is the power consumption of DC heating mode at the same heating voltage. It is worth mentioning that the P_average_ of the sensor was as low as 0.075 mW, which is one three-hundredth of that in the DC heating mode. The repeatability and long-term stability of sensor based on hierarchical In_2_O_3_ for NO_2_ under pulse heating was also measured as shown in Figure 8c,d. It can be seen that under the pulse-driven mode, the sensor displayed good reproducibility, reversible and stable ability. The result indicates that the MEMS NO_2_ sensor based on the hierarchical In_2_O_3_ can work at an ultra-low power consumption and obtain a high sensitivity and low LOD in the pulse-driven heating mode.

### 3.5. Parameters Comparison and Sensitive Mechanism

**Figure 9:**
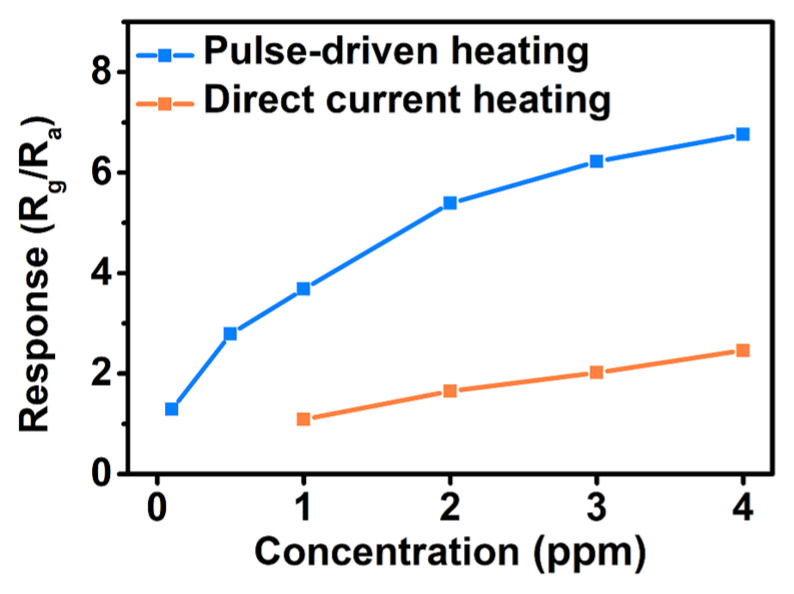
Sensitivities of the sensor based on hierarchical In_2_O_3_ to 1–4 ppm (DC heating mode) and 0.1–4 ppm (pulse-driven heating mode) of NO_2_.

The responses in different heating modes are given in Figure 9. Compared with DC heating modes, the sensor working in the pulse-driven heating mode showed the higher sensitivity. It is worth mentioning that the P_average_ of the sensor was as low as 0.075 mW, which was 1/300 of that in the DC heating mode. The result indicates that the MEMS NO_2_ sensor based on hierarchical In_2_O_3_ can work at an ultra-low power consumption and obtain a high sensitivity and low LOD in the pulse-driven heating mode.

**Figure 10:**
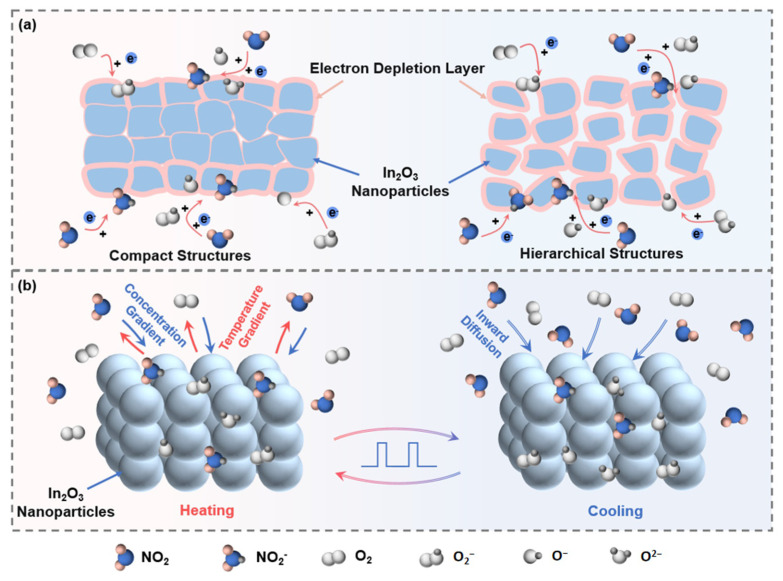
Schematic illustration: (**a**) sensing mechanism of sensors based on hierarchical In_2_O_3_; (**b**) gas molecular behavior under different stages.

The sensing mechanism was explored to explain the phenomenon described above. To better explain the gas sensing characteristics, a schematic diagram of sensing mechanism in different heating modes was established (Figure 10). The oxygen adsorption model was employed to explain the appearance of smaller R*_a_* in the pulse heating mode. In_2_O_3_ is a commonly used n-type MOS, and when oxygen molecules are adsorbed on the surface of In_2_O_3_, they will occupy the previously formed oxygen vacancies and take electrons from the material conduction band to form negative oxygen species. At low temperatures (T < 150 °C), the adsorbed oxygen on the surface of In_2_O_3_ is mainly O_2_^−^, as shown in following equation:

(2) $$O_{2}+e^{-}\to O_{2}^{-}$$

At high temperatures (150 °C < T < 400 °C), oxygen anions are mainly present in the form of O^−^ [43,44], as shown in following equation:

(3) $$O_{2}^{-}+e^{-}\to 2O^{-}$$

The temperature in the continuous heating mode was 160 °C, and the mainly oxygen anions were O^−^. The temperature in the pulse heating mode could reach 160 °C too, but it quickly fell back to room temperature. In the process of rapid heating and cooling, the reaction to produce chemical adsorbed oxygen was divided into two stages. The first stage was that the sensor temperature was below 150 °C, and the type of chemical adsorbed oxygen generated at this time was O^2−^. In the second stage, when the sensor was in the range of 150 to 160 °C, the type of chemical adsorption oxygen generated was O^−^. Due to the short time at high temperature, O^2−^ on the sensor surface could not be completely converted to O^−^. At this time, there were two kinds of adsorbed oxygen O^2−^ and O^−^. Since the formation of negative oxygen needed to consume electrons from the material conduction band, an electron depletion layer was formed on the In_2_O_3_ surface. In this work, the electron depletion layer (EDL) in the continuous heating mode was thicker than that in pulse mode, because more electrons had been taken from the material conduction band to form negative oxygen O^−^ which led the dozen times smaller R*_a_* and the performance of higher sensitivity characteristics in the pulse mode.

The reaction of NO_2_ on the material surface can be divided into physisorption and chemisorption which can be expressed, respectively, as follows [45,46]:

(4) $${NO}_{2}+O_{2}^{-}+2e^{-}\to {NO}_{2}^{-}+2O^{-}$$

(5) $${NO}_{2}+O^{-}+2e^{-}\to {NO}_{2}^{-}+O^{2-}$$

(6) $${NO}_{2}+e^{-}\to {NO}_{2}^{-}$$

In this research, flower-like In_2_O_3_ samples, which were made up of thin 2D sheets, were synthesized and the loose and porous hierarchical structure was designed. Incorporating hierarchical structures into sensing materials enhances the utility of the sensitive body, thereby enhancing the sensing performance in comparison with dense bulk materials [47,48]. The loose and porous form of the samples facilitated the penetration of gas molecules into the inside of the In_2_O_3_ materials (Figure 10a).

In addition, it is worth noting that the gas sensing characteristics were optimized in the pulse-driven heating mode compared with the DC heating mode. In the DC heating mode, continuous high operating temperatures inevitably promoted molecular desorption on the surface of the materials and reduced the concentration of surrounding target gas, as shown in Figure 10b [27,49]. In the pulse-driven heating mode, the low temperature stage reduced the desorption of the target gas [50]. As the temperature gradient disappeared, the gas concentration gradient drove the surrounding target gas to diffuse inside the sensing layer and conduct physical adsorption on the material’s surface to provide reactants for successive chemisorption and redox reaction in the next cycle’s T_on_ [51]. The above-mentioned process optimized the response and LOD of the sensors under the pulse-driven heating mode.

## 4. Conclusions

In this work, a hierarchical In_2_O_3_-based MEMS NO_2_ gas sensor was designed and fabricated. The temperature and gas sensitivity characteristics under pulse-driven with ultra-low power consumption were studied. The results show the sensor obtained the better gas sensing characteristics with pulse-driven pulse heating (T*_on_* = 0.1 s, T*_wait_* = 29.9 s) to NO_2_ detection ranging from 100 ppb to 4 ppm. The power consumption of the sensor was greatly reduced in the pulse-driven heating mode (0.075 mW), which was only 1/300 of the DC heating mode (22.5 mW). Furthermore, the gas sensing behaviors at different heating modes is also illustrated. This work provides a new idea and possibility for the application of the sensor in the field of wearable, portable devices and Internet of things (IoTs), where power consumption requirements are more stringent.

## Data Availability

Data are contained within the article.
